# Establishment of enzyme-linked immunosorbent assays based on recombinant S1 and its truncated proteins for detection of PEDV IgA antibody

**DOI:** 10.1186/s12917-022-03262-z

**Published:** 2022-04-27

**Authors:** Ying Shan, Qin Gao, Junyong Mao, Jingyou Zheng, Xiaohan Xu, Chuni Zhang, Xiaojun Huang, Jidong Xu, Fushan Shi, Min Yue, Fang He, Weihuan Fang, Xiaoliang Li

**Affiliations:** 1 Department of Veterinary Medicine, College of Animal Sciences, Zhejiang University, Hangzhou, 310058 Zhejiang China; 2 Present Address: Zhejiang Provincial Key Lab of Preventive Veterinary Medicine, MOA Key Laboratory of Animal Virology, Center for Veterinary Sciences, Zhejiang University, Hangzhou, 310058 Zhejiang China; 3 Present Address: Hainan Institute, Zhejiang University, Yongyou Industry Park, Yazhou Bay Sci-Tech City, 572000 Sanya, China

**Keywords:** PEDV, Bac-to-Bac Eukaryotic expression, Recombinant S1 truncated protein, ELISA

## Abstract

Porcine epidemic diarrhea virus (PEDV) can infect pigs of all ages, especially piglets. PEDV has spread across Asia since the 1980s. The highly virulent variant PEDV broke out on a large scale and caused huge economic losses to the pig industry in late 2010 in China. Rapid detection methods with high specificity and sensitivity are urgently needed for the diagnosis and control of the disease. In this study, we divided the PEDV S1 gene into three segments and constructed the recombinant plasmids pFastBac1-S1T1 (aa 21–279), pFastBac1-S1T2 (aa 280–539) and pFastBac1-S1T3 (aa 540–788), which carry the different antigenic regions of the S1 gene. Truncated S1 proteins PEDV-S1T1/S1T2/S1T3 were obtained by a Bac-to-Bac expression system, with protein sizes of 36 kDa, 38 kDa and 38 kDa, respectively. Recombinant proteins presented high reactivity with the monoclonal antibody against PEDV and positive pig serum. Based on full-length S1 protein and these truncated proteins, we established indirect ELISA methods for the detection of PEDV IgA antibody. A total of 213 clinical serum samples were tested by the above indirect ELISA methods, and IFA was used as the gold standard. ROC curves revealed a significant correlation between S1-ELISA and S1T2-ELISA with a 0.9134 correlation coefficient and favourable sensitivity and specificity of S1-ELISA (93.24%, 95.68%) and S1T2-ELISA (89.33%, 94.16%). Our results also indicated that serum with higher neutralizing activity (SNT ≥ 40) had a higher IgA antibody level based on S1-ELISA, S1T1-ELISA and S1T2-ELISA. In conclusion, both S1-ELISA and S1T2-ELISA can be used as candidate systems for detecting anti-PEDV IgA antibody titers in serum, which can reflect the level of neutralizing activity in pigs after natural infection or vaccination. The above research results provide a basis for the prevention and control of PEDV and can be used in the detection of host anti-infective immunity and evaluation of vaccine immune effects.

**Supplementary Information:**

The online version contains supplementary material available at 10.1186/s12917-022-03262-z.

## Introduction

Porcine epidemic diarrhea (PED) first appeared in the United Kingdom [1] and Belgium [2] in the early 1970s and then gradually spread to other European countries. After the 1990s, the disease showed sporadic local outbreaks in Europe [3]. Porcine epidemic diarrhea virus (PEDV) has gradually become popular in Asian countries since it was first reported in Asia in 1982. In October 2010, the highly virulent variant PEDV broke out on a large scale and then spread across the country in China [4]. Survey data showed that the detection rates of PEDV-positive samples ranged from 61.10% to 78.49%, while the rates of PEDV-positive pig farms ranged from 71.43% to 83.47% [5, 6]. Pigs of all ages are affected by PEDV, among which the mortality rate of suckling piglets is as high as 100% [4, 7, 8]. The high morbidity and mortality have brought huge economic losses to the pig industry.

PEDV is a member of the alpha coronavirus family with a particle approximately 95–190 nm in diameter [1] and has an envelope, a genome length of approximately 28 kb containing the 5' noncoding region (5'-UTR), a single-stranded positive-stranded RNA and a cap structure and a poly A tail in the 3'-end noncoding region (3'-UTR) [2]. The viral genome consists of a 5'-UTR, 3'-UTR and seven open reading frames (ORF1a, ORF1b, ORF2, ORF3, ORF4, ORF5 and ORF6). The replicase polyprotein 1ab (pp1ab) encoded by ORF1a and ORF1b is cleaved by papain (PL pro) and 3C-like protease (3CL pro) into 16 nonstructural proteins (Nsp1-16) [9, 10]; ORF2-6 encode ORF3 nonstructural proteins and four structural proteins, including spike protein (S protein), small membrane protein (E protein), membrane glycoprotein (M protein) and nucleocapsid protein (N protein) [11]. The PEDV S protein is divided into two domains according to its function: the N-terminal S1 subunit (aa 1–789) is responsible for recognizing cell surface receptors, and the C-terminal S2 subunit (aa 790–1383) is responsible for mediating virus and cell membrane fusion [12]. When the virus invades the cell, the S1 protein binds to the surface receptor of the host cell and induces the production of neutralizing antibodies; the S2 protein mediates the fusion of the virus with the host cell membrane, allowing the virus genome to enter the host cell [13].

Several studies have reported the establishment of ELISA detection methods for PEDV, which mainly focus on PEDV S protein, N protein and M protein. An ELISA method based on PEDV N protein were established to for serological screening [14]. M protein-based ELISA was developed for serological evaluation of PEDV [15]. Studies have found that S-ELISA is more sensitive than N-ELISA, and antibodies against S protein last longer in the body than N protein [16]. Moreover, these ELISA methods are developed to detect IgG against PEDV. Secretory immunoglobulin A (IgA), which can neutralize viruses and prevent viruses from invading the intestinal epithelium, plays an important role in intestinal mucosal immunity [17]. IgA can be detected in serum and colostrum of pigs after virus challenge or inoculation, and therefore serum and colostrum IgA antibodies to enteric pathogens can act as indicaStors [18–20]. Therefore, the establishment of IgA ELISA based on S protein will have a valuable effect and application prospect. Considering about the large molecular size and complex structure of S protein, some important epitopes will be hidden inside, thus portion of S protein, mostly S1 protein, has been reported as antigens for ELISA establishment [21]. However, the effect of IgA ELISAs based on different truncated S1 proteins was neither compared in contrast with that of S1 based ELISA, nor analyzed the co-relations with neutralization activity. In this study, we described an indirect ELISA method against PEDV IgA with high sensitivity and specificity. S1 and its three truncated proteins were used as coating antigens to explore the accuracy of ELISA. IgA antibody levels detected by S1-ELISA and S1T2-ELISA, which were correlated to neutralization activity, can be used to evaluate the immunity state of PEDV infected or vaccinated pigs.

## Methods

### Strains and plasmids

*E. coli* DH5α and DH10Bac were cultured in Luria broth (LB) medium in a rotatory incubator (150 rpm) at 37 °C. DH5α was used to preserve the positive clones in the process of plasmid construction. DH10Bac was used to generate the recombinant bacmid. The pFastBac™ vector (Life Technologies, USA) was used as the backbone vector to construct the recombinant expression plasmids.

The PEDV S1 gene was analyzed by the transmembrane area prediction server (http://sbcb.bioch.ox.ac.uk/TM_noj/TM_noj.html) and divided into three segments: S1T1, S1T2 and S1T3. The primers used are listed in Table 1. The three segments were amplified from the PEDV S1 gene fragment, which was modified based on the codon usage bias of the SF9 cell line. The truncated S1 fragments were then fused with melittin signal peptides at the N terminal and 6 × His tags at the C terminal. The modified truncated S1 fragments were inserted into the pFastBac™ vector between the EcoRI and HindIII restriction endonuclease sites and named pFastBac-S1T1, pFastBac-S1T2 and pFastBac-S1T3.

**Table 1:** Primers used in this study

Primer	Nucleotide sequence (5' to 3')	Product Length (bp)
1-F	CCGGAATTCATGAAATTCTTAGTCAACGTTGCCCTTG	90
1-R	AGAACCGCTGCCGGAACCC	
2-S1T1-F	GGGTTCCGGCAGCGGTTCTCAAGATGTGACTCGTTGCTCCGC	777
2-S1T1-R	CAGCAAGGGTTGGTTGCTGACC	
2-S1T2-F	GGGTTCCGGCAGCGGTTCTGTCAACTGTCTCTTGGCTATCCCTAAGATC	780
2-S1T2-R	CGAGAAGCCGTTGATAGTGGTGTCA	
2-S1T3-F	GGGTTCCGGCAGCGGTTCTTCCTTCTGTGTCGATACCCGCC	747
2-S1T3-R	TGAGAAGTTGGTCGGGATGGAGAT	
3-S1T1-F	GGTCAGCAACCAACCCTTGCTGGGTTCCGGCAGCGGTTCTCA	57
3-S1T2-F	TGACACCACTATCAACGGCTTCTCGGGTTCCGGCAGCGGTTCTCA	
3-S1T3-F	ATCTCCATCCCGACCAACTTCTCAGGTTCCGGCAGCGGTTCTCA	
3-R	CCCAAGCTTTTAGTGATGGTGATGGTGGTGATGATG	

### Cells and virus

The SF9 insect cell line, cultured in SF900III medium (Lifetechnology, USA) supplemented with antibiotics consisting of 0.1 mg/mL streptomycin, 100 units/mL penicillin, and 0.25 μg/mL amphotericin B at 28 °C in a rotatory incubator (120 rpm), was used to obtain recombinant S1 truncated protein. The Vero E6 cell line, cultured in DMEM with the antibiotics mentioned above and 10% fetal bovine serum (FBS, Gibco, USA), was used for virus propagation.

The PEDV epidemic strain ZJ15XS0101 was kept in our lab. Recombinant baculovirus with high viral titers was generated in recombinant bacmid-transferred SF9 cells after two rounds of amplification.

### Serum and antibody

Different positive sera were used to detect the specificity of ELISA. Classical swine fever virus (CSFV)-, porcine circovirus type 2 (PCV2)-, porcine reproductive and respiratory syndrome virus (PRRSV)-positive serum samples, and PEDV reference positive and negative sera were prepared and kept by our lab. Transmissible gastroenteritis virus (TGEV)-positive serum was kindly provided by Hunan University.

A total of 213 pig blood samples were collected from Aug 2013 to Apr 2019 from local farms in Zhejiang Province. Serum was separated from blood samples by centrifugation at 1500 × g for 10 min and kept at -20 °C until use.

Previously produced mouse anti-S1 monoclonal antibody 6B4/6G1/1C11 could recognize S1T1, S1T2, S1T3, respectively. PEDV positive serum and mouse anti-His monoclonal antibody (Beyotime, China) were used as primary antibody. HRP-labeled goat anti-mouse IgG antibody (Beyotime, China). HRP-labeled goat anti-pig IgG antibody (Lifetechnology, USA), Alexa Fluor 488-conjugated goat anti-pig IgA antibody (Lifetechnology, USA) and Alexa Fluor 568-conjugated goat anti-mouse IgG antibody (Lifetechnology, USA) were used as secondary antibody.

### Eukaryotic expression and purification of recombinant S1 truncated protein

Recombinant plasmids containing S1, S1T1, S1T2 and S1T3 were respectively transformed into *E. coli* DH10Bac. After cultured for 12–14 h, the positive recombinant bacmids were generated which were screened by blue-white selection and verified by PCR. The recombinant bacmids were then transfected into SF9 cells by using Lipofectamine 2000 (Invitrogen, USA). Viral supernatant from positive wells were collected and stored at -80 °C. For each sample, the positive virus was amplified and the virus titer was determined for the following infection. Finally, the truncated S1 recombinant proteins were expressed in the amplified recombinant baculovirus infected SF9 cells with the MOI of 0.1. The supernatants containing truncated S1 proteins were then separated from cell cultures at 72 h post infection. For purification, 200 mL supernatant was added to Ni–NTA column (Yeasen, China). After washing with 10 mM imidazole, the purified truncated S1 recombinant protein was eluted with 5 mL of 400 mM imidazole and stored at -80 °C.

### Recombinant S1 truncated protein identification

Recombinant proteins were identified by immunofluorescence assay in SF9 cells as well as Coomassie Blue staining and western blot analysis of purified proteins. Briefly, SF9 cells transfected with recombinant bacmids for 72 h were fixed in 4% paraformaldehyde at 4 °C overnight. After washing by 1 × PBS (0.01 M pH 7.2), the fixed cells were permeabilized with 0.2% Triton X-100 at room temperature for 15 min and blocked with PBS containing 10% BSA for 1 h. The samples were then incubated with mouse anti-His monoclonal antibody (dilution 1:1000) as primary antibodies and Alexa Fluor 568-Goat anti-mouse IgG (dilution 1:1000) as the secondary antibody, each at 37 ℃ for 1 h. Cell nuclei were stained with DAPI (Beyotime, China) at a 1:1000 dilution. Immunofluorescences were observed by using a laser confocal microscope IX81-FV1000 (Olympus, Japan).

Coomassie Blue staining and western blot analysis methods were carried out to furtherly verify the protein expression. Briefly, gels containing protein samples were separated by SDS-PAGE, stained with Coomassie Blue for 3 h at 37 °C and then decolorized by destaining solution (5% ethanol and 10% acetic acid glacial) overnight. For Western blotting, the parallel gels were electrotransferred onto a polyvinylidene difluoride membrane (Millipore, Billerica, MA). The membranes were blocked for 1 h in Tris-buffered saline containing 0.05% Tween 20 (TBST) and 5% skimmed milk followed by probing overnight at 4 °C with primary and secondary antibodies. Mouse anti-His monoclonal antibody, mouse anti-S1 monoclonal antibody 6B4/6G1/1C11 strain, and PEDV-positive serum were used as primary antibodies (dilution 1:2000) respectively, and HRP-labeled goat anti-mouse IgG antibody and HRP-labeled goat anti-pig IgG antibody were used as secondary antibodies (dilution 1:2000). The results were visualized using West Pico chemiluminescent substrate (Thermo, Marina, CA) under the conditions recommended by the manufacturer. Images were captured in a Gel 3100 chemiluminescent imaging system (Sagecreation, Beijing, China).

### PEDV indirect fluorescent antibody (IFA) test

PEDV indirect fluorescent antibody test was carried to identify the positive sample. Briefly, after infection of PEDV for 16 h, cells were fixed, permeabilized and blocked as described before. The samples were then reacted with different pig sera as the primary antibodies (dilution 1:200) at 37 ℃ for one hour and then labeled with Alexa Fluor 488 goat anti-pig IgA antibody as the secondary antibody (dilution 1:1000) at 37 ℃ for 1 h. Each serum was tested by IFA three times, and positive and negative serum controls were included throughout the experiment. Cell nuclei were stained with DAPI (Beyotime, China) at a 1:1000 dilution. Images were visualized using a laser confocal microscope IX81-FV1000 (Olympus, Japan). PEDV reference positive and negative sera were used as positive and negative controls.

### ELISA

Indirect ELISA assays were carried out and optimized with porcine serum samples. Briefly, 96-well microplates were coated overnight at 4 °C with purified S1 and its truncated proteins diluted in 100 mM carbonate-bicarbonate buffer (A500873, Sangon Bitech, China, pH 9.6). After washing 3 times with PBS, the wells were blocked with solution reagents containing 0.05 g/mL sucrose (A502792, Sangon Biotech, China), 0.1% proclin-300 (48,912, Sigma, USA) and 0.02 g/mL BSA (C510069, Sangon Biotech, China) in 10 mM PBS at 37 °C. After three washes with PBST (0.01 M pH 7.2 with 0.5% Tween-20), 100 μL of serum from ZJ15XS0101-infected pigs diluted in PBST-5% milk was added into each well and incubated at 37 °C. After washing 3 times, the reaction mixture was then incubated with HRP-labeled goat anti-pig IgA antibody (dilution, 1:1500). After incubation and subsequently washing, the color was developed by the addition of 100 μL of substrate solution consisting of tetramethylbenzidine chromogenic substrate (Sigma-Aldrich) and stopped by the addition of 50 μL of 2 M H_2_SO_4_. The optical densities at 450 nm (OD_450nm_) were measured in a microplate reader (BioTek, USA). Each serum sample was tested in triplicate, each well was read twice, and the cutoff point of a positive sample was set to be at least twice the negative sample at any dilution. Different antigen concentrations, serum dilutions and incubation times of each step were tested by checkboard assays to optimize the ELISA method.

### SNT

The pig serum samples were inactivated at 56 °C for 30 min and diluted by a factor of 2, which began at 1:5. The virus at 10^4^ TCID_50_/mL with diluted serum in the same volume was mixed and incubated at 37 °C for 2 h. Vero-E6 cells were inoculated into 96-well plates and cultured to 90–95% confluency. Then, the medium was replaced with neutralized virus. A total of 4 parallel wells were set for each serum dilution sample. Serum and virus were used as the negative and positive controls, respectively. Viruses were removed after 2 h of absorption. The cells were washed twice with Hank’s solution before maintenance medium (DMEM with 8 μg/mL trypsin) was added. The CPE was determined 3 days post infection. The serum neutralization titer was calculated according to the Reed-Muench method.

### Statistical analysis

GraphPad Prism (GraphPad Software, USA) was used to analyze the data and the statistical significance was tested by one-way analysis of variance (one-way ANOVA). All results were presented as the mean ± SE of triplicate experiments.

## Results

### Eukaryotic expression of S1 truncated protein by the Bac-to-Bac system

**Fig. 1:**
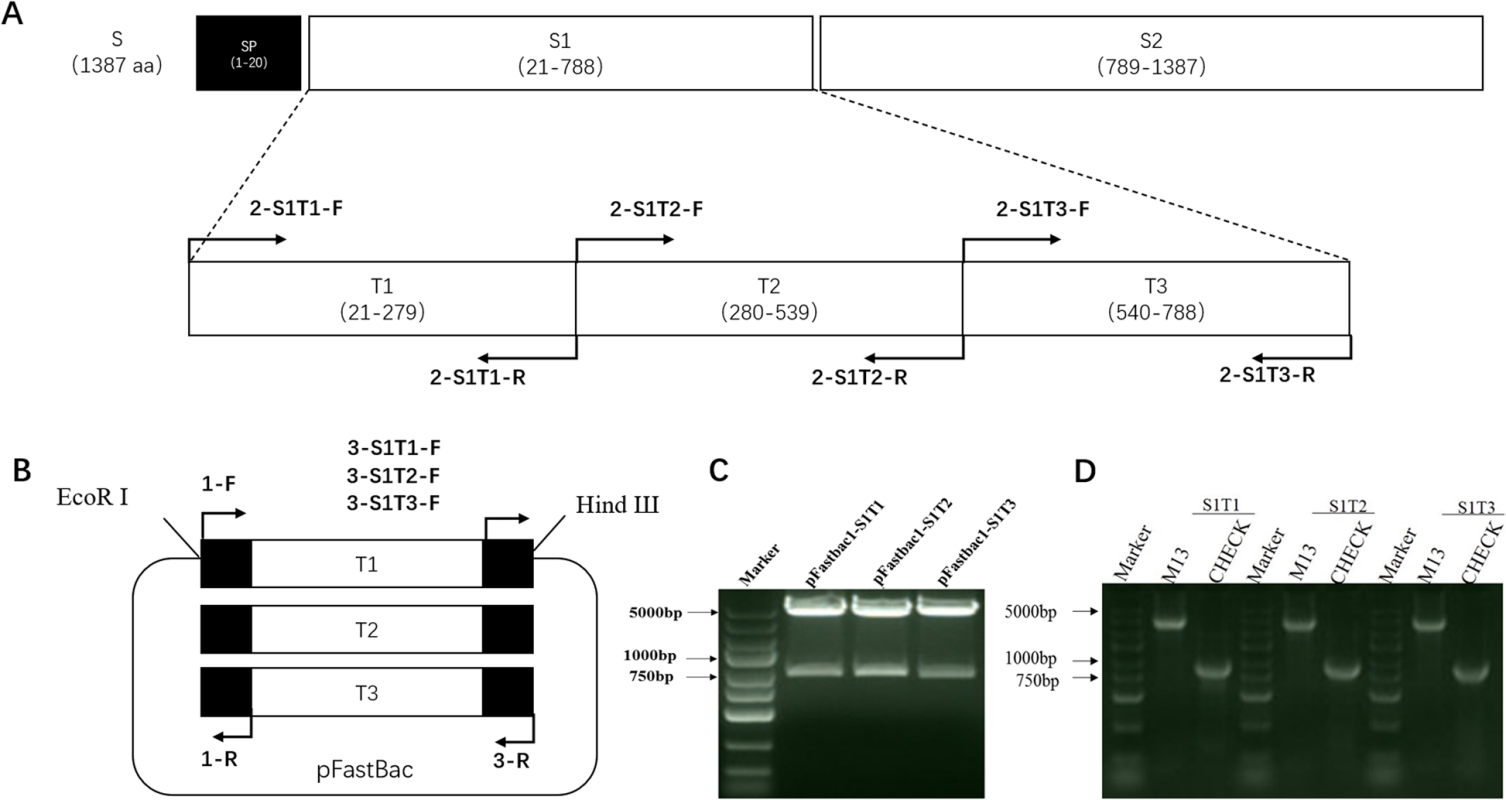
The construction of S1T1, S1T2 and S1T3 recombinant balculovirus.** A** Division strategy of PEDV S1 gene into three segments as S1T1, S1T2 and S1T3. **B** Construction strategy of S1T1, S1T2 and S1T3 recombinant balculovirus plasmids. **C** Identification of recombinant balculovirus plasmids by double digestion identification with target fragments of 4827 bp (pFASTBac plasmid), 924 bp (S1T1), 927 bp (S1T2) and 894 bp (S1T3). **D** Identification of the recombinant bacmids by PCR with expected products respectively

**Fig. 2:**
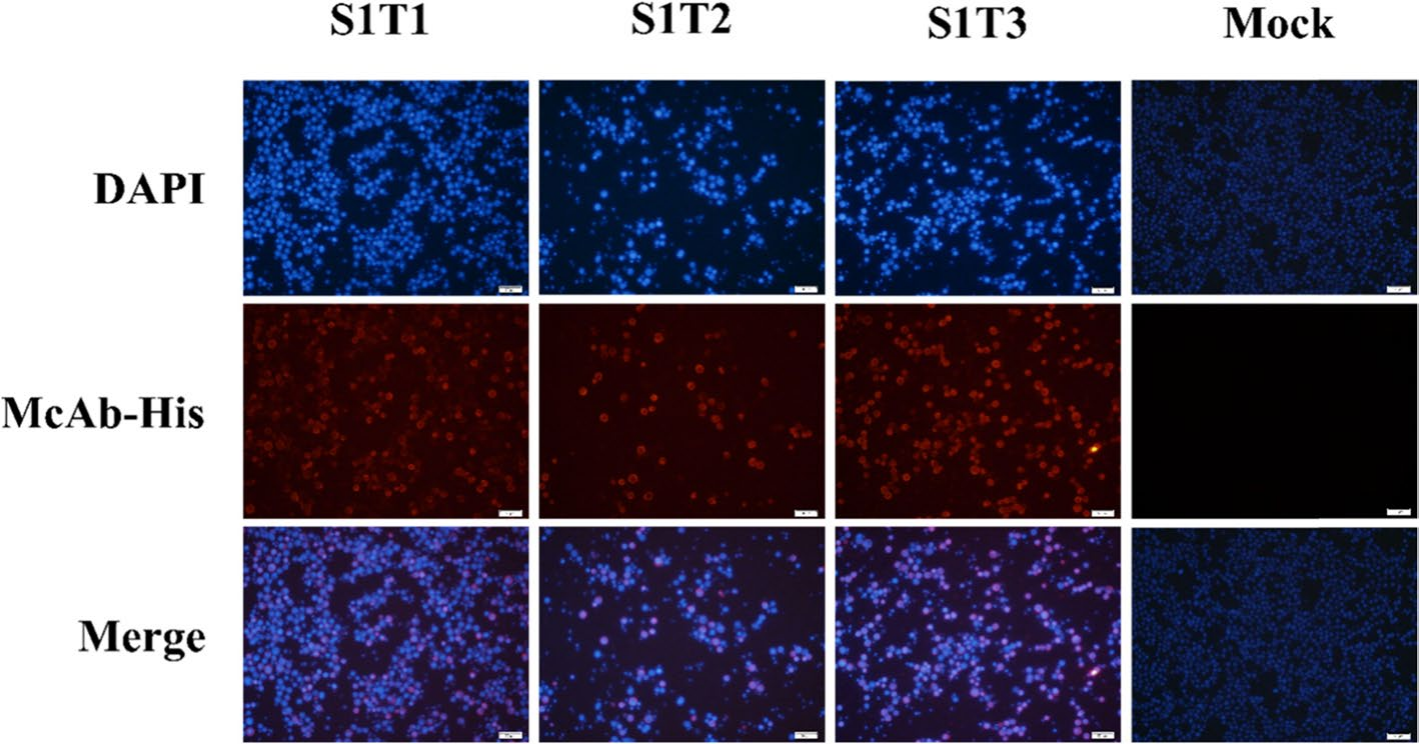
Identidication of the recombinant baculovirus infection by immunofluoresence assay. Baculovirus infections were identified by immunofluoresence using his tag monoclonal antibody. Positive cells (red) with recombinant S1T1/S1T2/S1T3 expression were observed in infected cells

**Fig. 3:**
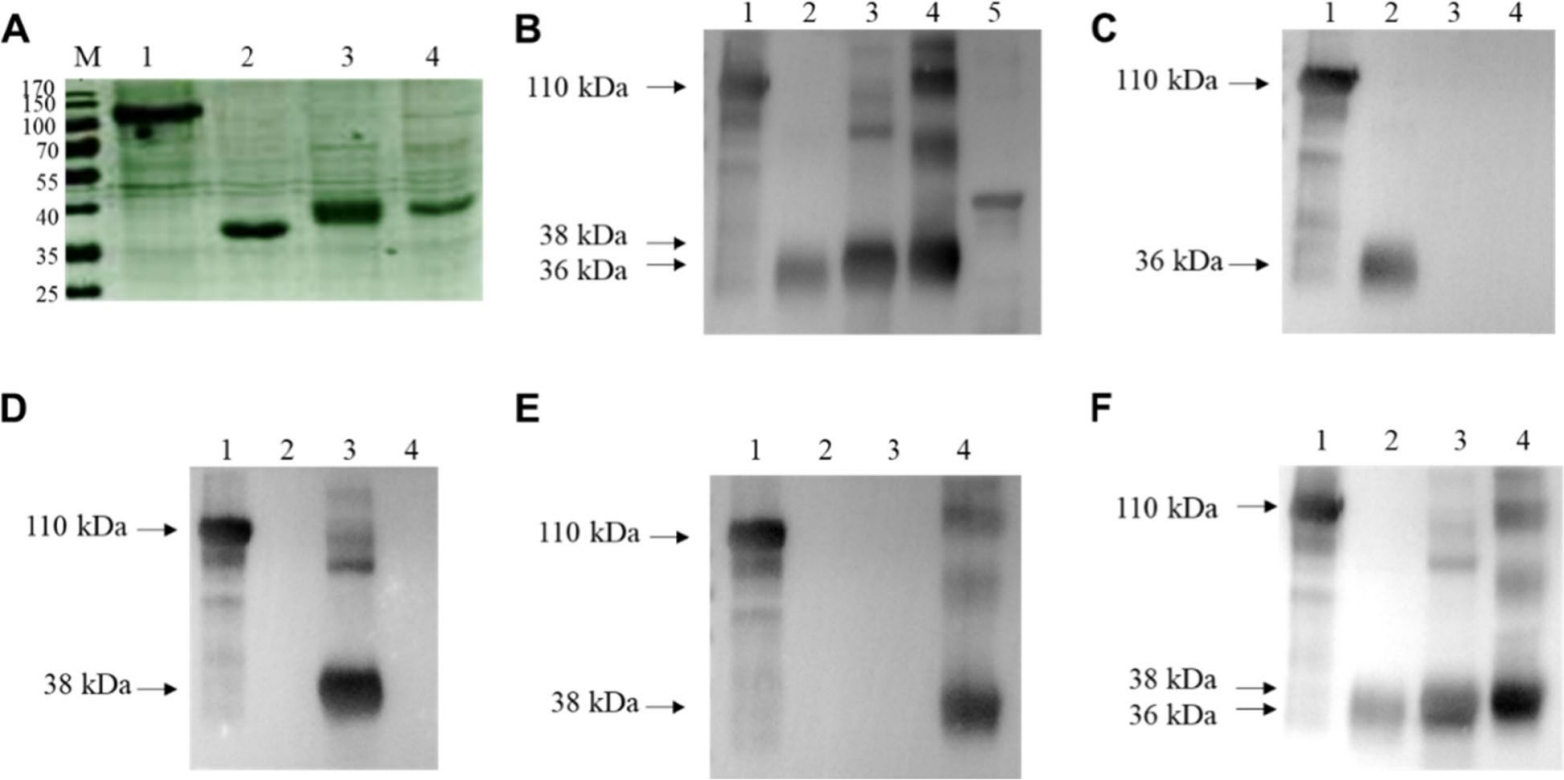
Identification of recombinant S1 truncated protein expression. **A** SDS-PAGE of S1 and its truncated fragments with Coomassie blue staining; **B** western blotting analysis of the S1 and its truncated fragments with anti-His McAb; **C** western blotting analysis of S1T1 with anti-PEDV S1 McAb-6B4; **D** western blotting analysis of S1T2 with anti-PEDV S1 McAb-6G1; **E** western blotting analysis of S1T3 with anti-PEDV S1 McAb-1C11; **F** western blotting analysis of S1 protein and its truncated fragments with PEDV positive pig serum. (Lane1: PEDV-S1; Lane2: PEDV-S1T1; Lane3: PEDV-S1T2; Lane4: PEDV-S1T3; Lane5: His tag positive control)

Previously, we established an S1-ELISA method against PEDV IgG [22]. Based on this method, we replaced IgG antibody with IgA antibody in secondary antibody incubation so that we established the S1-ELISA method against PEDV IgA. To further improve the specificity of the ELISA detection method, we designed an optimization strategy with truncated S1 protein (Fig. 1A and B). The recombinant bacmids containing S1T1, S1T2 and S1T3 were successfully constructed and identified by plasmid double digestion identification with target fragments of 4827 bp (pFASTBac plasmid), 924 bp (S1T1), 927 bp (S1T2) and 894 bp (S1T3). We also identified the recombinant bacmids by PCR using M13 primers and specific primers with the expected products (Fig. 1C and D). The recombinant baculovirus was generated successfully. Cytopathic effects (CPE), such as cell swelling and abscission, were observed in infected cells (Fig. S1). Baculovirus infections were also identified by immunofluoresence using a tag antibody (Fig. 2). Recombinant S1 truncated proteins were identified by SDS-PAGE (Fig. 3A) with Coomassie blue staining and western blotting analysis using a His-tagged antibody (Fig. 3B). We also tested the reactivities of the S1 monoclonal antibody and the recombinant truncated proteins. Monoclonal antibodies 6B4, 6G1 and 1C11 can identify S1T1, S1T2, and S1T3, respectively (Fig. 3C-E). PEDV-positive serum was also reactive with these truncated proteins (Fig. 3F).

### Development of ELISA based on recombinant S1 truncated protein

**Fig. 4:**
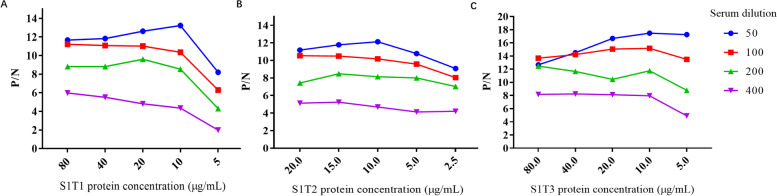
Optimization of coating antigen concentration and serum dilution of truncated S1 ELISA.** A** S1T1 ELISA was optimized by different antigen coating concentration (80, 40, 20, 10 and 5 μg/mL) and serum dilution (1:50, 1:100, 1:200 and 1:400); **B** S1T2 ELISA was optimized by different antigen coating concentration (20, 15, 10, 5 and 2.5 μg/mL) and serum dilution (1:50, 1:100, 1:200 and 1:400); **C.** S1T3 ELISA was optimized by different antigen coating concentration (1:50, 1:100, 1:200 and 1:400 μg/mL) and serum dilution (1:50, 1:100, 1:200 and 1:400)

The optimal working concentration of antigen coating and the appropriate dilutions of sera were confirmed to be 10 μg/mL and 1:50 for all three truncated proteins, which were determined by checkerboard assays using serial dilutions of antigens and sera (Fig. 4). The conditions of ELISA, such as antigen coating, antibody incubation and substance reaction, were optimized under different temperatures and times (data not shown). High P/N value can be obtained while antigen was coated at 4℃ for 14 h, antibody was incubated at 37℃ for 1 h and substance reaction was at 37℃ for 10 min. The S1/1S1T1/S1T2/S1T3-ELISA against IgA showed no cross reactivities with CSFV-, PCV2-, PRRSV- and TGEV-positive serum (Table 2).

**Table 2:** Specificity of PEDV S1/S1T1/S1T2/S1T3 ELISA

Serum	PEDV	CSFV	PCV2	PRRSV	TGEV
**IgA ELISA value**					
** S1T1**	1.3190 (+)	0.0763 (-)	0.1921 (-)	0.0536 (-)	0.0852 (-)
** S1T2**	1.2917 (+)	0.0143 (-)	0.0177 (-)	0.0085 (-)	0.0266 (-)
** S1T3**	1.4191 (+)	0.1287 (-)	0.0079 (-)	0.0453 (-)	0.0964 (-)
** S1**	0.9372 (+)	0.0585 (-)	0.0474 (-)	0.0066 (-)	0.0756 (-)

### ROC analysis of ELISA

**Fig. 5:**
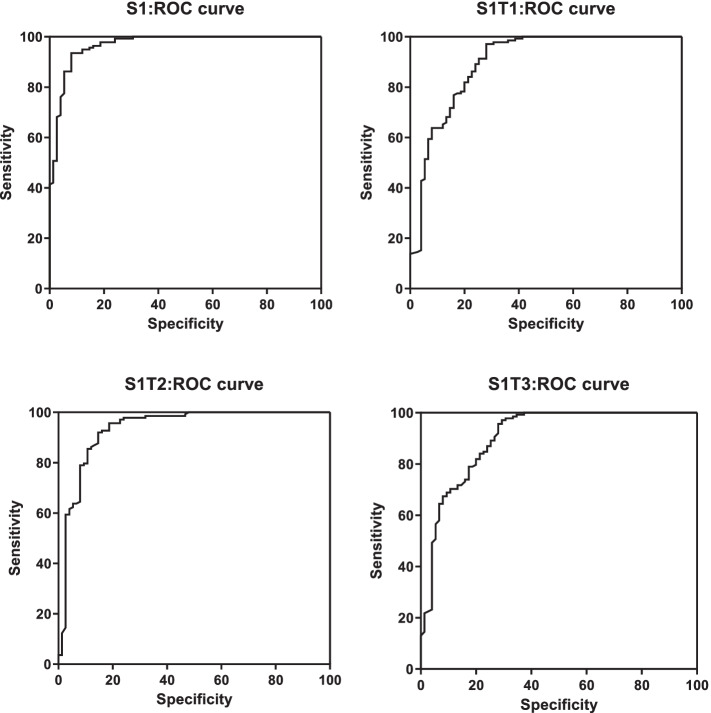
ROC analysis of truncated S1 ELISA. A total of 213 pig serum samples were tested for the presence of anti-PEDV IgA by S1/S1T1/S1T2/S1T3-ELISA and by IFA. The sensitivity/specificity and the area under curve (AUC) of S1/S1T1/S1T2/S1T3-ELISA was determined by ROC analysis taking IFA results as standard

In 213 pig serum samples, 75 (35.21%) and 138 (64.79%) were determined to be PEDV IgA positive and negative, respectively. Typical IgA IFA results are shown in Fig. S2. Taking the IFA results as gold standard, the sensitivity and specificity of ELISA were analyzed by the ROC curves. The ROC analysis data are shown in Fig. 5 and Table 3 with the cutoff value, sensitivity and specificity, the area under the curve (AUC), and the 95% confidence interval of S1/S1T1/S1T2/S1T3-ELISA. The S1/S1T1/S1T2/S1T3-ELISA against IgA results presented 78/59/65/70 positive samples and 135/154/138/143 negative samples, containing 9/4/10/13 false-positive and 6/20/10/18 false-negative samples, respectively (Fig. S3). Together, these results indicate that the S1-ELISA and S1T2-ELISA against IgA presented high relative sensitivity and relative specificity.

**Table 3:** ROC results of PEDV S1/S1T1/S1T2/S1T3 ELISA

	**S1**	**T1**	**T2**	**T3**
**cut-off**	0.4129	0.7102	0.3311	0.3707
**Sensitivity (%)**	93.48	97.1	92.75	90.58
**Specificity (%)**	92	73.33	86.67	76
**AUC**	0.9683 ± 0.0110	0.9001 ± 0.0248	0.9392 ± 0.020	0.9044 ± 0.0234
**95% confidence interval**	0.9449 to 0.9916	0.8515 to 0.9487	0.8999 to 0.9785	0.8585 to 0.9504
***P* value**	< 0.0001	< 0.0001	< 0.0001	< 0.0001

### IgA antibody by ELISA could reflect the neutralization antibody titer

**Fig. 6:**
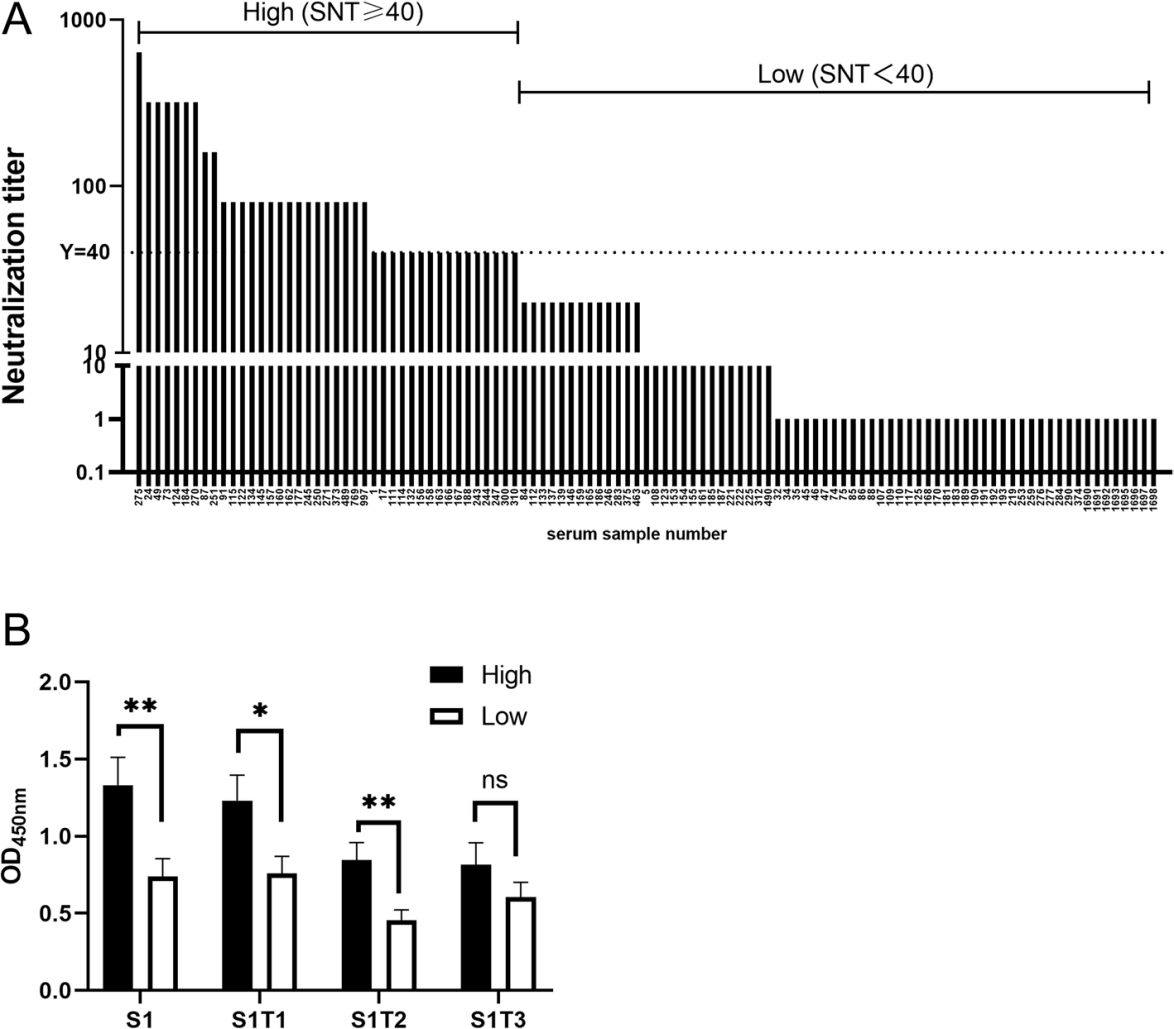
Relevance between S1T1/S1T2/S1T3-ELISA value with serum neutralization titer.** A** A total of 109 pig serum samples were tested for serum neutralization and were divided into two group. High group was SNT ≥ 40 and low group was SNT < 40; **B** A total of 109 pig serum samples were tested for the presence of anti-PEDV IgA by S1T1/S1T2/S1T3-ELISA. The relevance between the results of S1/S1T1/S1T2/S1T3-ELISA against PEDV IgA with SNT. The IgA ELISA OD_450nm_ value of serum with high neutralization antibody titer was significantly higher than that with low neutralization antibody titer. *: *P* < 0.5; **: *P* < 0.05; ns: no significance

We tested 109 samples of pig serum collected from local farms in Zhejiang Province. Serum was divided into two groups: a high group with SNT ≥ 40 and a low group with SNT < 40 (Fig. 6A).

A total of 109 pig serum samples were tested for the presence of PEDV IgA by S1/S1T1/S1T2/S1T3-ELISA and tested for the neutralization antibody titer. The ELISA results showed significant relevance to the serum neutralization titer (Fig. 6B). The ELISA OD_450nm_ value of serum with a high neutralization antibody titer was significantly higher than that with a low neutralization antibody titer. These results indicate that our ELISA methods based on S1 and its truncated proteins could reflect the neutralization capability of the serum sample.

## Discussion

As a porcine intestinal disease, PED causes very similar clinical symptoms to other intestinal coronavirus diseases, such as TGEV and porcine deltacoronavirus (PDCoV). Therefore, laboratory tests are necessary to confirm the diagnosis. Serological testing can not only achieve rapid detection but also monitor the changes in antibody levels in pigs, which could be used to evaluate the immune effect after vaccination. ELISA is the most commonly used serological test method for commercial or laboratory use. Commercial PEDV detection ELISA kits are usually targeted at N protein, which is massively produced protein in infection and is able to induce strong immune response [14, 23]. However previous study reported there was cross reactivity in PEDV N protein based ELISA with other swine coronavirus [24]. It is also demonstrated that PEDV S-ELISA was more sensitive than N-ELISA, and antibodies against S protein last longer in the body than that against N protein [16]. The good immunogenicity and reactogenicity of the S protein make it an ideal target protein for PEDV detection. Most of the identified epitopes are located in the S1 region [25]. The large molecular size of S protein made it difficult in large scale expression, many studies have also used S1 instead of full-length S protein to establish serological detection methods and prepare vaccines [26, 27].

Since the S protein displays a complex structure and contains N-glycosylation sites [28], here we applied a baculovirus expression system for the acquisition of S1 and its truncated proteins. The baculovirus expression system, as a widely used and highly efficient eukaryotic system, allows for natural conformation of complex multidimensional structures and post-translational modifications, and allows the insertion of larger exogenous DNA fragments. Credit to the signal peptide genetic modification, it is easy to obtain proteins from the culture supernatants, which were of high purity and large amounts for ELISA antigen coating. Therefore, S1 and its truncated proteins generated from this optimized baculovirus expression system can present large quantity, good immunogenicity and high sensitivity for ELISA methods.

In this study, we compared the sensitivity and specificity of ELISA based on S1 and its truncated proteins. The ROC curve is used as a tool to evaluate the diagnostic value of the ELISA methods by calculating the area under the curve [29]. The Youden index is often used as the cut-off for negative and positive sera. In general, the larger the area under the curve, the higher the Youden index, and the more reliable the established detection method [30]. In order to measure the accuracy of our established methods, we used IFA results as the standard to analyze the indirect ELISA detection methods for anti-PEDV IgA antibody based on S1 and its truncated protein. These IgA ELISA methods all showed significant *P* values of ROC analysis with all AUCs above 0.90 as shown in Fig. 5, indicating excellent detection accuracy. Interestingly, the S1T2-ELISA had better performance compared to others truncated S1 ELISA methods while the S1-ELISA was superior on sensitivity and specificity in these ELISA methods possibly due to the more binding sites compare with other truncated proteins and the good immunogenicity obtained from the baculovirus expression system.

A few ELISA methods had been reported to detect IgA antibodies against PEDV and be applied to clinical diagnosis or vaccine effect evaluation. However, there is no more information on the correlation between neutralization activity and ELISA detection based on recombinant S1 and its truncated proteins for clinical serum samples. Our results demonstrated that the S1-ELISA, S1T1-ELISA and S1T2-ELISA results were closely correlated with their neutralization test results. Considering about the sensitivity and specificity, S1-ELISA and S1T2-ELISA against IgA could be used as candidate systems for detecting the neutralization titer of anti-PEDV IgA antibodies. PEDV mainly destroys intestinal epithelial cells, causing diarrhea, dehydration and mass death in suckling piglets. In vaccinated and infected pigs, IgG and IgA collectively contribute to the neutralizing antibody titer [31]. The specific IgA antibody taken from colostrum is the main antibody for suckling piglets to resist enteric swine coronavirus infection. It is reported that induced IgA antibody in sow milk can provide protection against virus to suckling piglets [32]. IgA, which can be transfer from sow serum to colostrum [33], could reflect the neutralization capability of colostrum [32]. Vaccination is widely used for the prevention and control of PEDV in China. Therefore, the serum IgA antibody ELISA detection methods established in this study can be used for not only PEDV infection detection but also vaccine immunity evaluation. In addition, it can also detect the IgA antibody in colostrum to predict the protection of piglets. The antibody level detected from these methods could also be regarded as an indicator for supplementary immunization indicator for sows.

Taken together, the established S1-ELISA and S1T2-ELISA against PEDV IgA could be used as a convenient tool to detect serum IgA antibody level reflecting the neutralization capability, as well as provide a basis for studying IgA antibody dynamic to PEDV infection and assessing the efficacy of vaccination.

## Supplementary Information

**Additional file 1:** **Fig. S1.** Identification of therecombinant baculovirus infection by cytopathic effects. The recombinant baculovirus were generated successfully. Cytopathic effects (CPE) such as cell swelling was observed in infected cells. **Fig. S2.** Representative results of immunofluorescence. Immunofluorescence assays were performed on PEDV infected cells. Serums were used as primary antibodies, while PBS was used in mock control. Alexa Fluor 488-conjugated goat anti-pig IgA antibody was used as secondary antibody. Outof 213 pig serum samples from local farms, 75 (35.21%) and 138 (64.79%) were shown to be PEDV IgA positive and negative, respectively. Typical fluorescence was presented. The nuclei were stained blue with DAPI; PEDV infected cells were stained with green fluorescence by positive serum, andsyncytia was visible. **Fig. S3.** ROC data distribution. Serum samples that positive in both ELISA and IFA were regard as true-positive; Serum samples that positive in ELISA but negativein IFA were regard as false-positive; Serum samples that negative in ELISA butpositive in IFA were regard as false-negative; Serum samples that negative in both ELISA and IFA were regard as true-negative. The cutoff values of S1/S1T1/S1T2/S1T3-ELISA against PEDV IgA were presented as dotted lines.

**Additional file 2.** 

**Additional file 3.** 

**Additional file 4.** 

**Additional file 5.** 

**Additional file 6.** 

**Additional file 7.** 

## Data Availability

The datasets generated and analyzed during the current study are available from the corresponding author on reasonable request.
