# Supplementary material for: Establishment of enzyme-linked immunosorbent assays based on recombinant S1 and its truncated proteins for detection of PEDV IgA antibody
Source: BMC Vet Res. 2022 Apr 27;18:154. doi: 10.1186/s12917-022-03262-z (PMC9043509; doi:10.1186/s12917-022-03262-z)
Supplement: Supplementary file 1 — Additional file 1: Fig. S1. Identification of therecombinant baculovirus infection by cytopathic effects. The recombinant baculovirus were generated successfully. Cytopathic effects (CPE) such as cell swelling was observed in infected cells. Fig. S2. Representative results of immunofluorescence. Immunofluorescence assays were performed on PEDV infected cells. Serums were used as primary antibodies, while PBS was used in mock control. Alexa Fluor 488-conjugated goat anti-pig IgA antibody was used as secondary antibody. Outof 213 pig serum samples from local farms, 75 (35.21%) and 138 (64.79%) were shown to be PEDV IgA positive and negative, respectively. Typical fluorescence was presented. The nuclei were stained blue with DAPI; PEDV infected cells were stained with green fluorescence by positive serum, andsyncytia was visible. Fig. S3. ROC data distribution. Serum samples that positive in both ELISA and IFA were regard as true-positive; Serum samples that positive in ELISA but negativein IFA were regard as false-positive; Serum samples that negative in ELISA butpositive in IFA were regard as false-negative; Serum samples that negative in both ELISA and IFA were regard as true-negative. The cutoff values of S1/S1T1/S1T2/S1T3-ELISA against PEDV IgA were presented as dotted lines. [file 12917_2022_3262_MOESM1_ESM.docx]

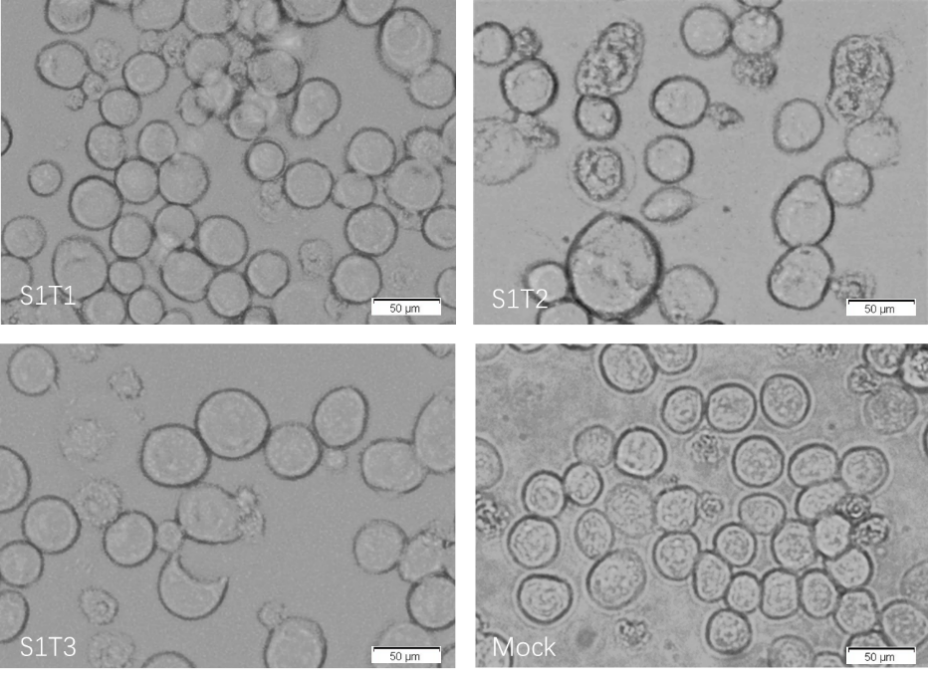


**Fig. S1 Identification of the recombinant baculovirus infection by cytopathic effects.** The recombinant baculovirus were generated successfully. Cytopathic effects (CPE) such as cell swelling was observed in infected cells.


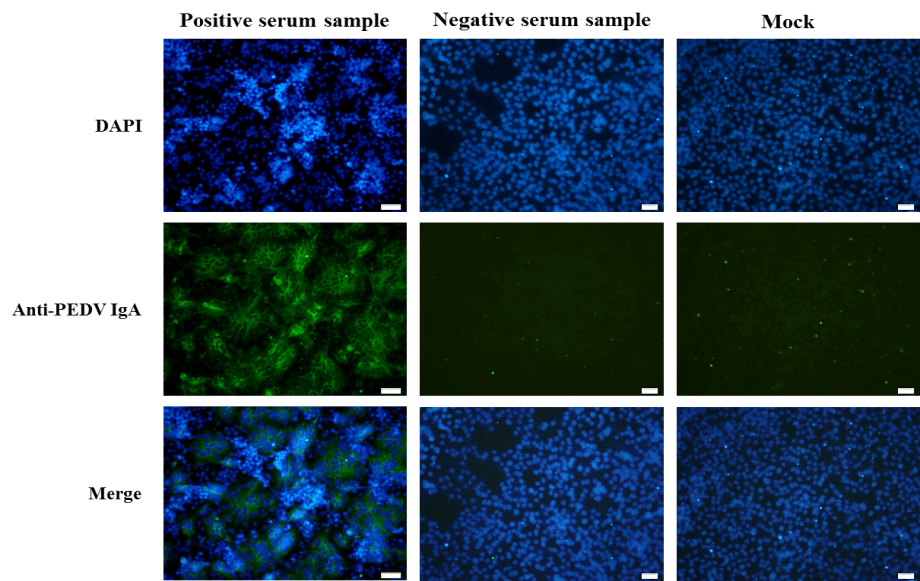


**Fig. S2 Representative results of immunofluorescence.** Immunofluorescence assays were performed on PEDV infected cells. Serums were used as primary antibodies, while PBS was used in mock control. Alexa Fluor 488-conjugated goat anti-pig IgA antibody was used as secondary antibody. Out of 213 pig serum samples from local farms, 75 (35.21%) and 138 (64.79%) were shown to be PEDV IgA positive and negative, respectively. Typical fluorescence was presented. The nuclei were stained blue with DAPI; PEDV infected cells were stained with green fluorescence by positive serum, and syncytia was visible.

**
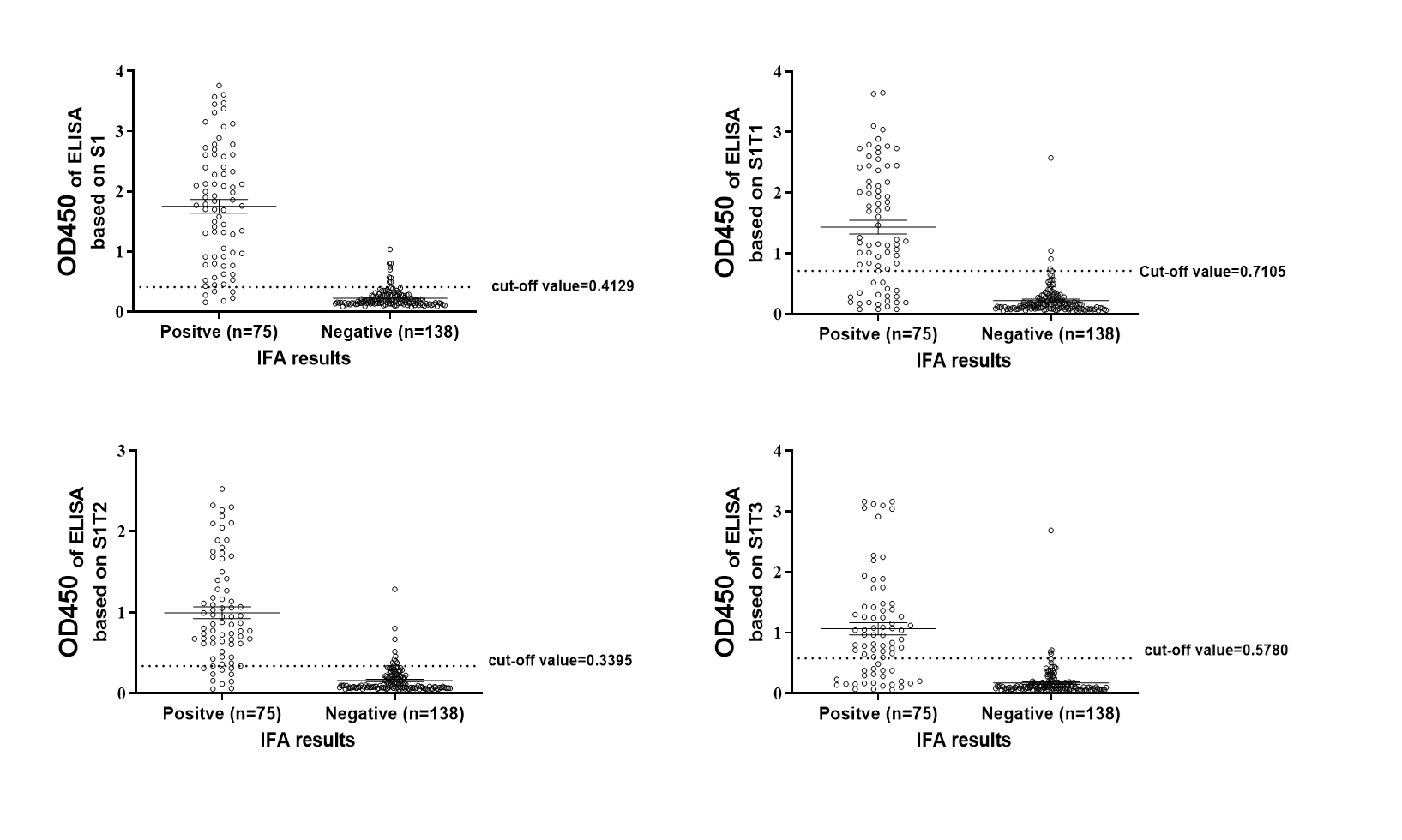
**

**Fig. S3 ROC data distribution.** Serum samples that positive in both ELISA and IFA were regard as true-positive; Serum samples that positive in ELISA but negative in IFA were regard as false-positive; Serum samples that negative in ELISA but positive in IFA were regard as false-negative; Serum samples that negative in both ELISA and IFA were regard as true-negative. The cutoff values of S1/S1T1/S1T2/S1T3-ELISA against PEDV IgA were presented as dotted lines.
