# Supplementary figures and images for: Establishment of enzyme-linked immunosorbent assays based on recombinant S1 and its truncated proteins for detection of PEDV IgA antibody
Source: BMC Vet Res. 2022 Apr 27;18:154. doi: 10.1186/s12917-022-03262-z (PMC9043509; doi:10.1186/s12917-022-03262-z)

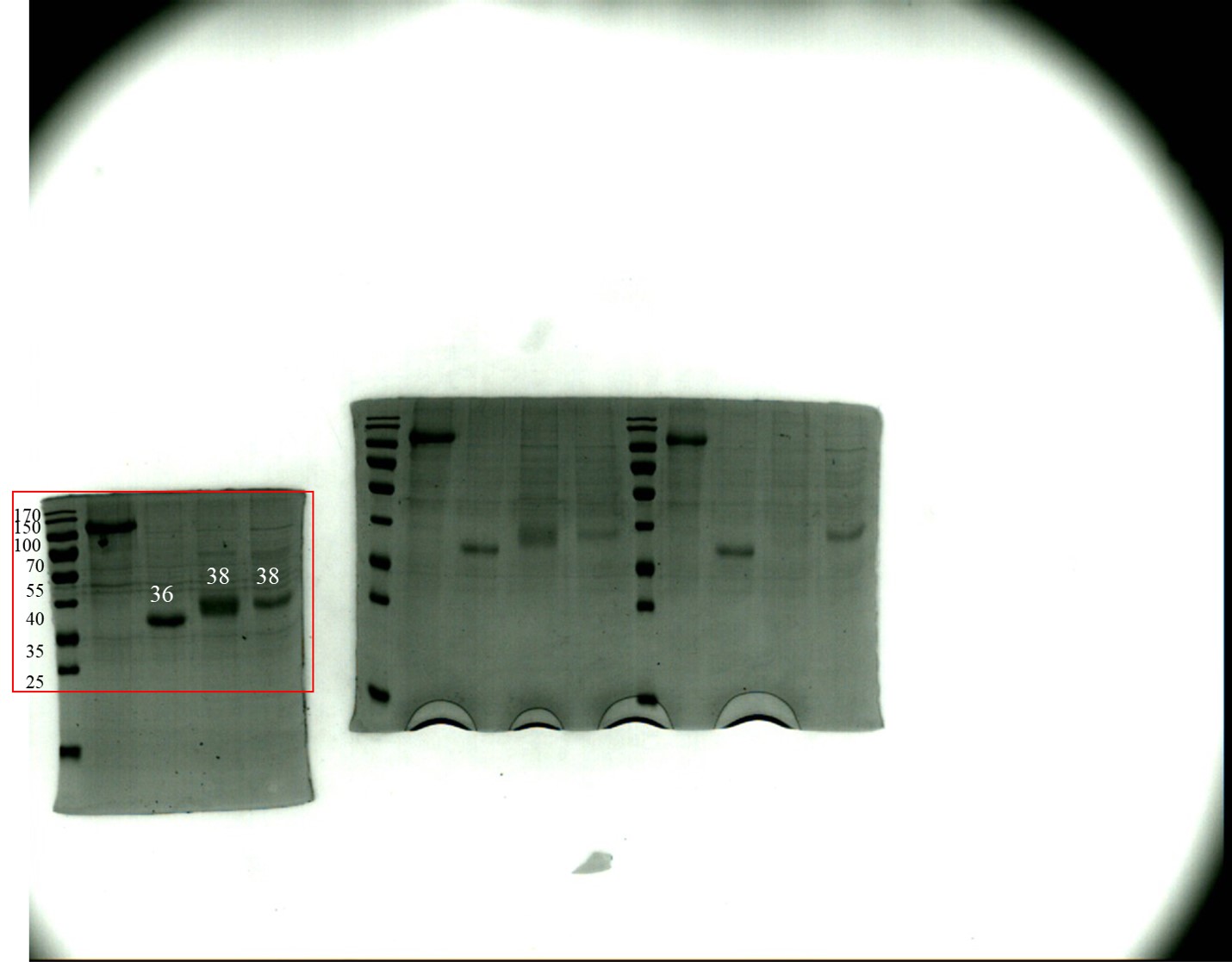

Supplement: Supplementary file 2 — Additional file 2. [file 12917_2022_3262_MOESM2_ESM.jpg]

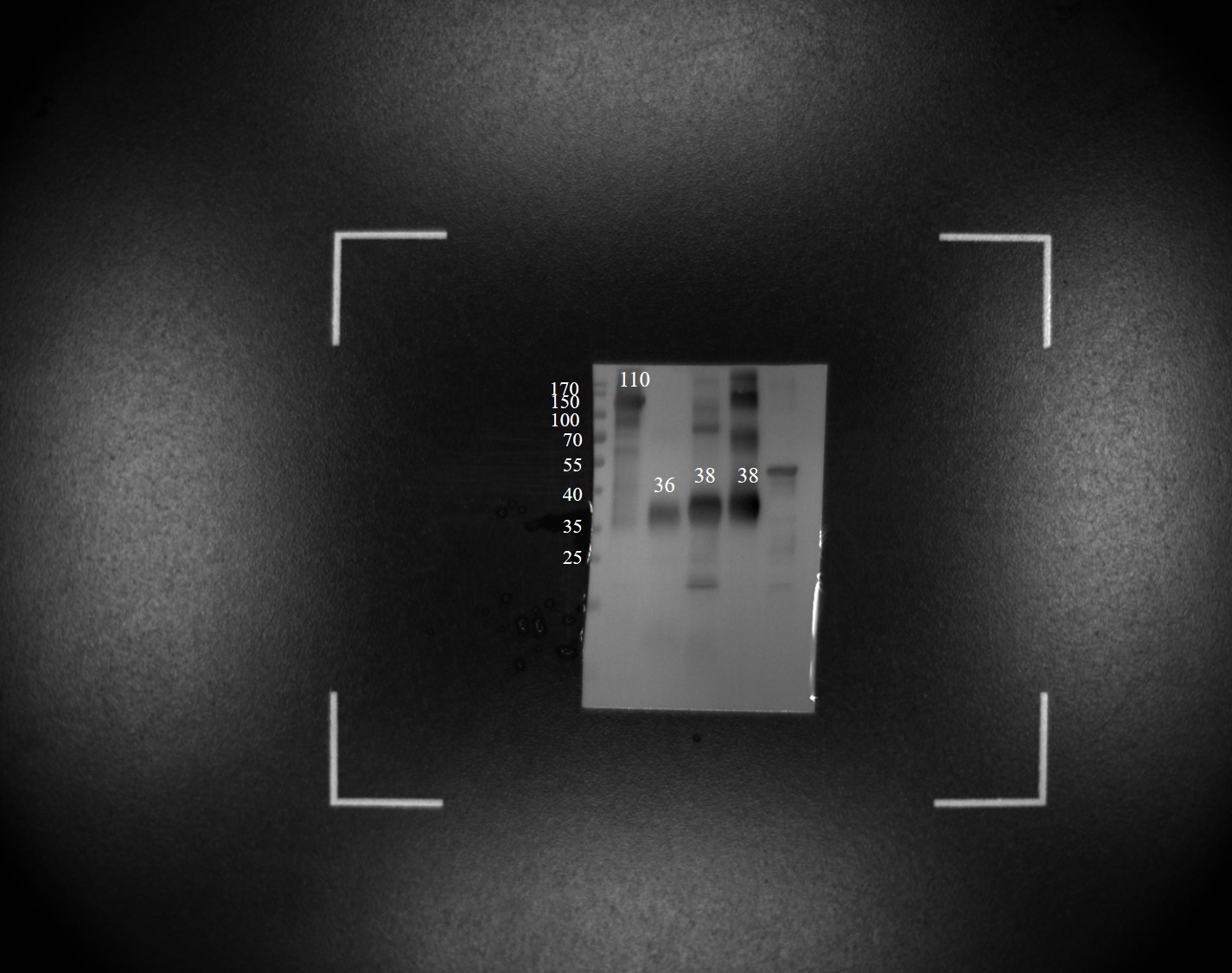

Supplement: Supplementary file 3 — Additional file 3. [file 12917_2022_3262_MOESM3_ESM.jpg]

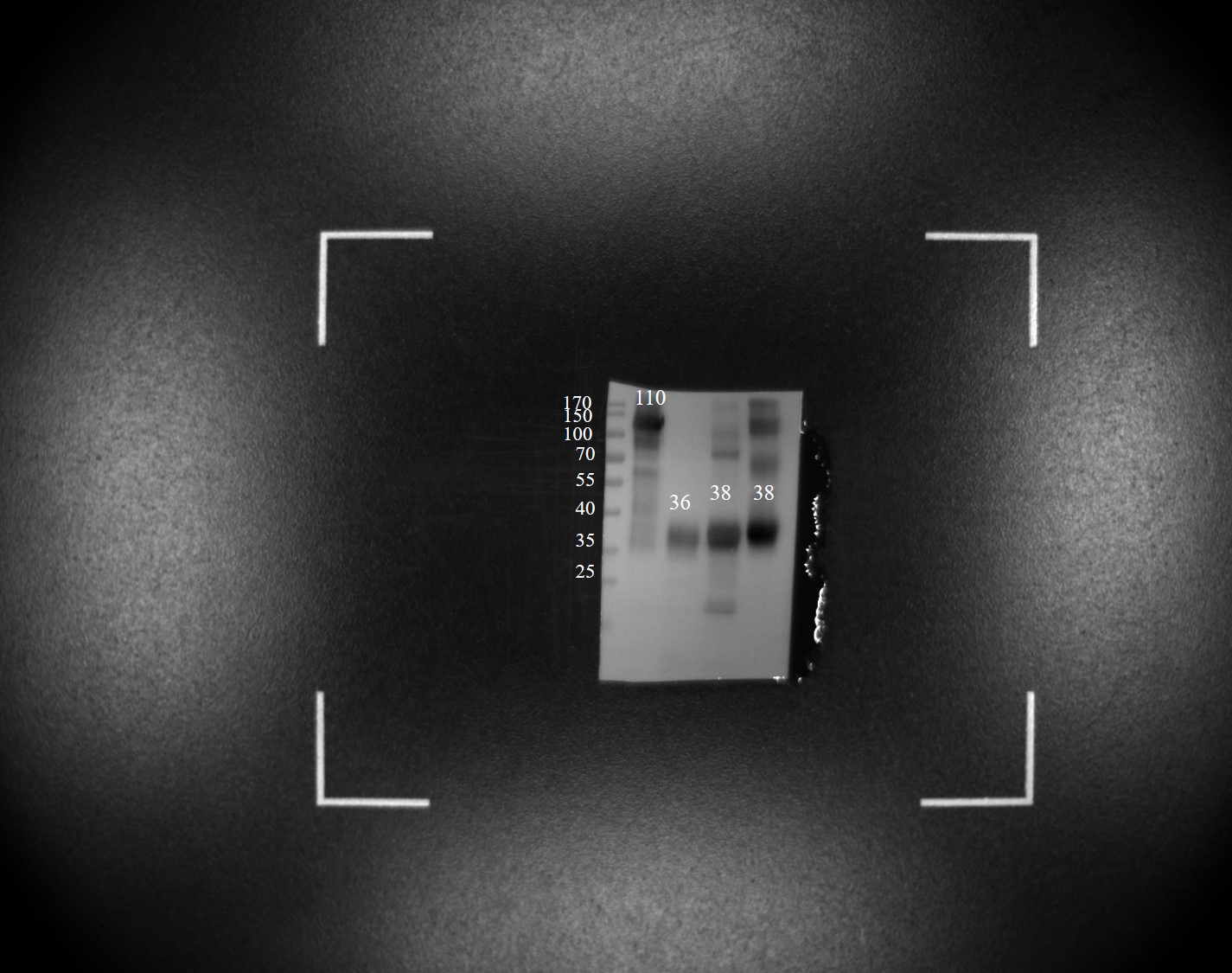

Supplement: Supplementary file 4 — Additional file 4. [file 12917_2022_3262_MOESM4_ESM.jpg]

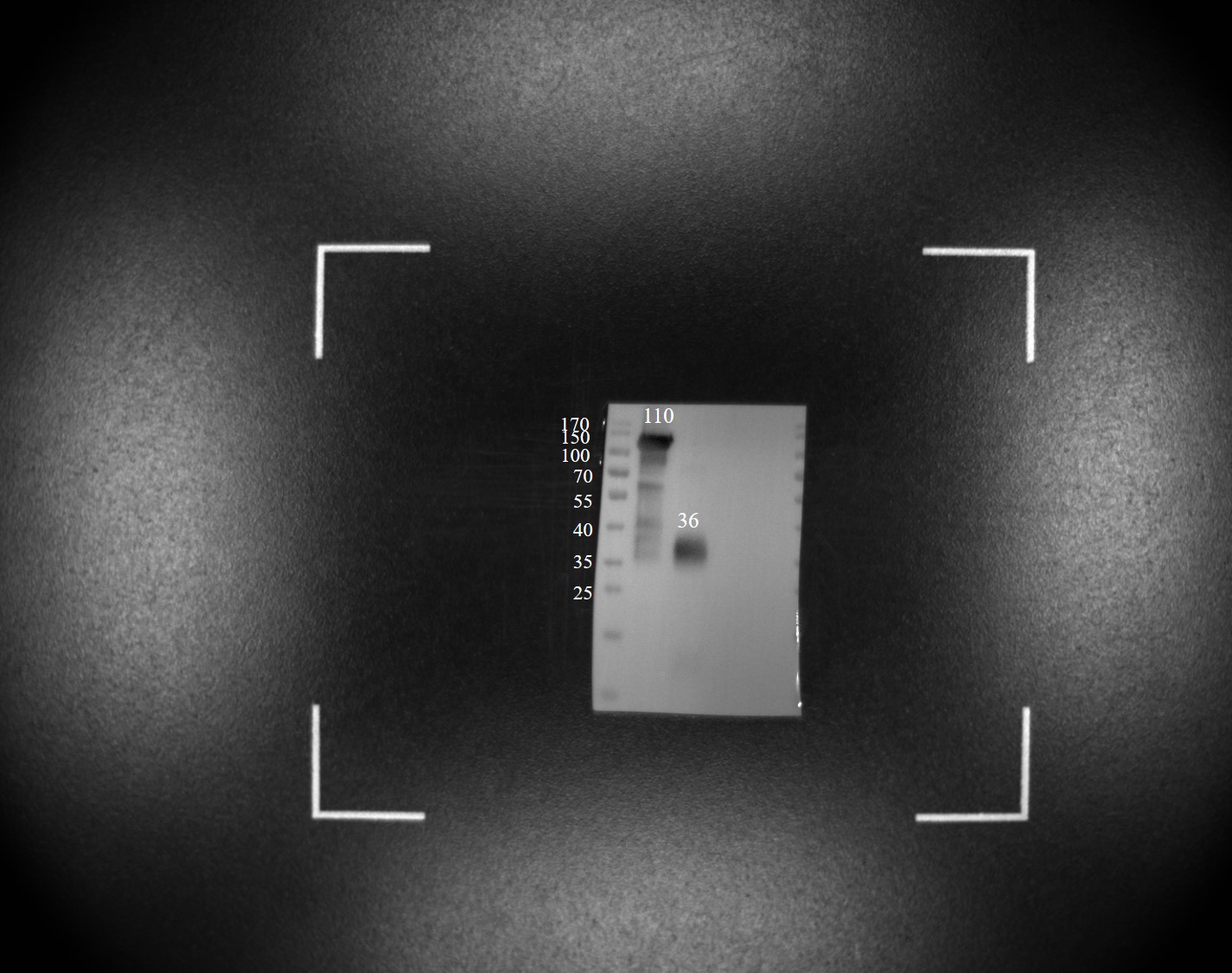

Supplement: Supplementary file 5 — Additional file 5. [file 12917_2022_3262_MOESM5_ESM.jpg]

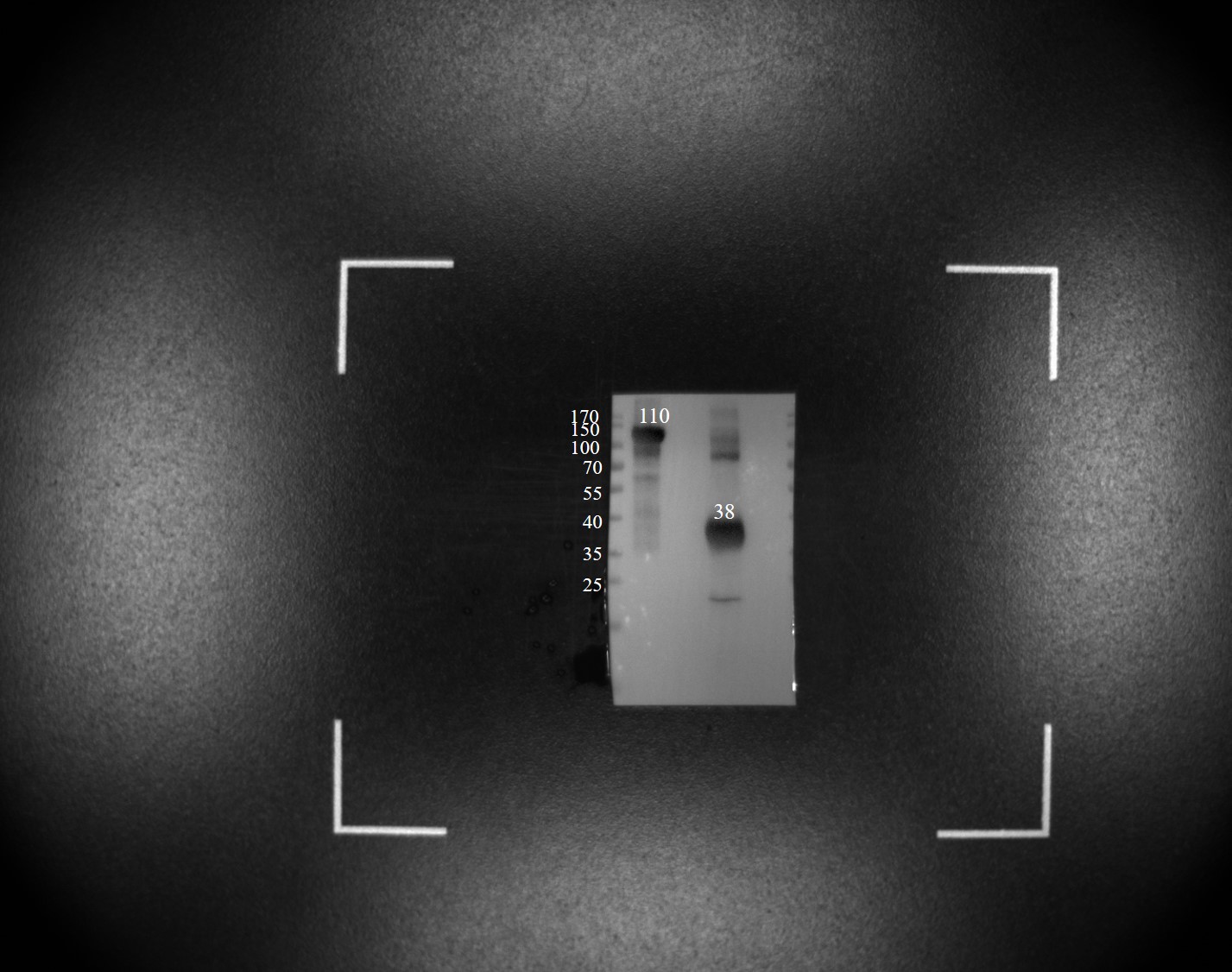

Supplement: Supplementary file 6 — Additional file 6. [file 12917_2022_3262_MOESM6_ESM.jpg]

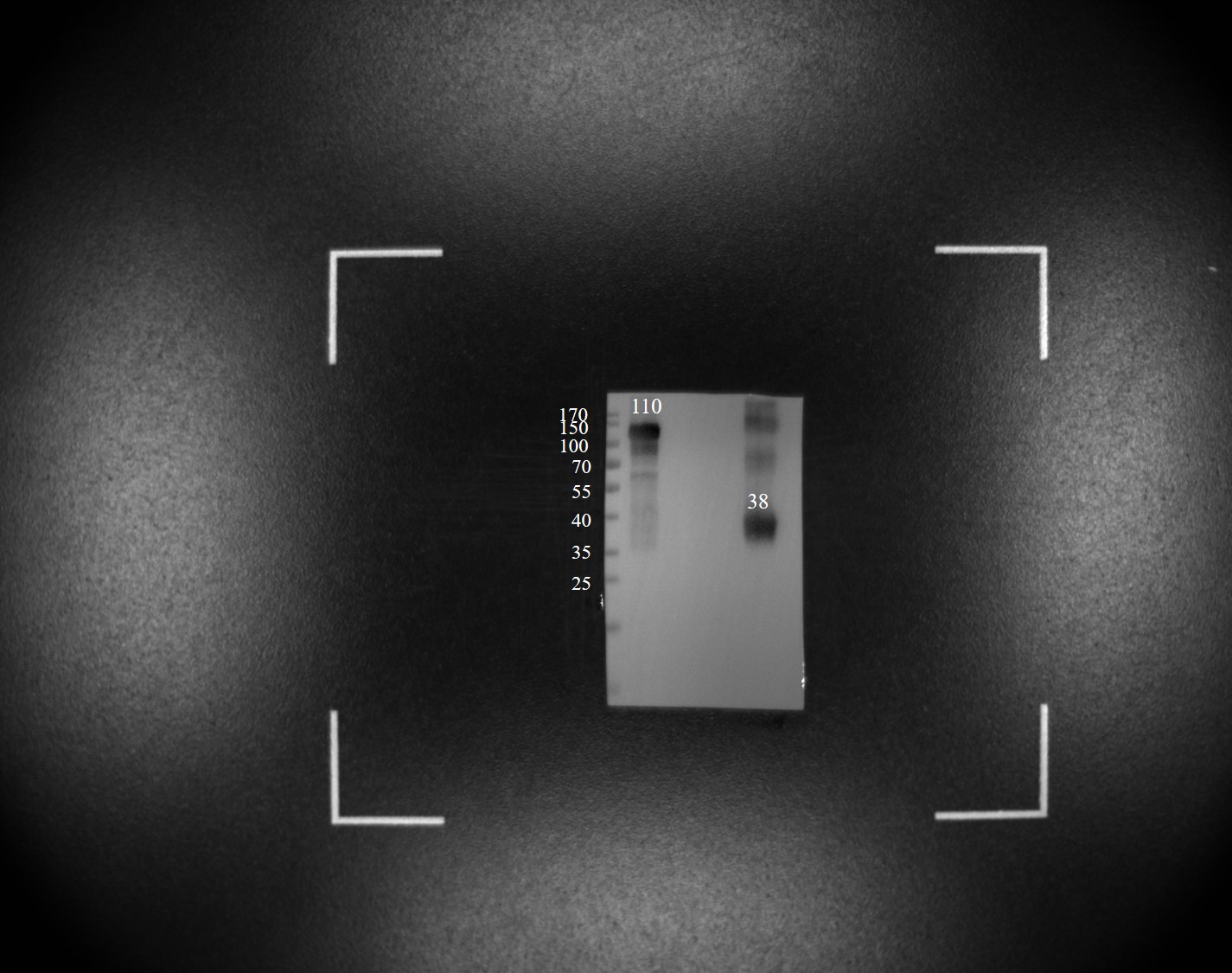

Supplement: Supplementary file 7 — Additional file 7. [file 12917_2022_3262_MOESM7_ESM.jpg]
